# Gut microbiome is linked to functions of peripheral immune cells in transition cows during excessive lipolysis

**DOI:** 10.1186/s40168-023-01492-3

**Published:** 2023-03-03

**Authors:** Fengfei Gu, Senlin Zhu, Yifan Tang, Xiaohan Liu, Minghui Jia, Nilusha Malmuthuge, Teresa G. Valencak, Joseph W. McFadden, Jian-Xin Liu, Hui-Zeng Sun

**Affiliations:** 1 Institute of Dairy Science, College of Animal Sciences, Zhejiang University, Hangzhou, 310058 China; 2 Ministry of Education Key Laboratory of Molecular Animal Nutrition, Zhejiang University, Hangzhou, 310058 China; 3 Agriculture and Agri-Food Canada, Lethbridge Research and Development Centre, 5403 1 Ave S, Lethbridge, AB T1J 4B1 Canada; 4 Department of Animal Science, Cornell University, 507 Tower Rd, Ithaca, NY 14850 USA

**Keywords:** Bile acids, Gut microbiome, Single-cell RNA sequencing, Targeted metabolomics

## Abstract

**Background:**

Postpartum dairy cows experiencing excessive lipolysis are prone to severe immunosuppression. Despite the extensive understanding of the gut microbial regulation of host immunity and metabolism, its role during excessive lipolysis in cows is largely unknown. Herein, we investigated the potential links between the gut microbiome and postpartum immunosuppression in periparturient dairy cows with excessive lipolysis using single immune cell transcriptome, 16S amplicon sequencing, metagenomics, and targeted metabolomics.

**Results:**

The use of single-cell RNA sequencing identified 26 clusters that were annotated to 10 different immune cell types. Enrichment of functions of these clusters revealed a downregulation of functions in immune cells isolated from a cow with excessive lipolysis compared to a cow with low/normal lipolysis. The results of metagenomic sequencing and targeted metabolome analysis together revealed that secondary bile acid (SBA) biosynthesis was significantly activated in the cows with excessive lipolysis. Moreover, the relative abundance of gut *Bacteroides sp. OF04 − 15BH*, *Paraprevotella clara*, *Paraprevotella xylaniphila*, and *Treponema sp. JC4* was mainly associated with SBA synthesis. The use of an integrated analysis showed that the reduction of plasma glycolithocholic acid and taurolithocholic acid could contribute to the immunosuppression of monocytes (CD14^+^MON) during excessive lipolysis by decreasing the expression of *GPBAR1*.

**Conclusions:**

Our results suggest that alterations in the gut microbiota and their functions related to SBA synthesis suppressed the functions of monocytes during excessive lipolysis in transition dairy cows. Therefore, we concluded that altered microbial SBA synthesis during excessive lipolysis could lead to postpartum immunosuppression in transition cows.

Video Abstract

**Supplementary Information:**

The online version contains supplementary material available at 10.1186/s40168-023-01492-3.

## Background

The transition period (3 weeks before and after parturition) is a critical stage of life for dairy cows because of excessive adipose tissue lipolysis that develops with reduced feed intake and an increased energy requirement to support lactation [1]. Excessive lipolysis generally develops with immunosuppression, which increases susceptibility to infections/diseases such as mastitis, metritis, and metabolic diseases [2]. These health issues are regarded as major management and economic challenges for the dairy industry that can also influence the long-term milk production performance of cows. Excessive lipolysis in transition cows is a multifactorial condition, in which both host metabolism and immune functions are altered [3, 4]. However, there is a lack of understanding of associative and causative relationships between host metabolism and immune regulation in transition dairy cows that experience elevated lipolysis. Therefore, a holistic approach to incorporate all the components of the lipolytic state is necessary to uncover mechanisms behind the altered metabolism and immune regulation of the transition cow.

Past studies have revealed that excessive lipolysis suppressed immune functions in transition dairy cows. For example, Cheng et al. [5] reported an inhibition in cell-to-cell adhesion in peripheral blood mononuclear cells (PBMCs) of cows with excessive lipolysis. Moreover, migration and chemotaxis of neutrophils (NEU) were suppressed in transition cows experiencing excessive adipose tissue lipolysis [6, 7]. These studies suggest that there is the degree of immunosuppression is directly related to the magnitude of body fat mobilization in transition dairy cows. However, we do not understand how the functionality of key immune cells, that are involved in immunosuppression, are impacted during excessive lipolysis. Single-cell RNA-sequencing (scRNA-seq) is a powerful tool that can be used to generate a comprehensive and precise landscape of immune cells [8, 9]. Profiling of immune cell transcriptomes using scRNA-seq will provide in-depth knowledge of the functionality of various cell populations and their roles in the immunosuppression during excessive lipolysis.

Studies on host-microbial interactions in humans and mice have revealed that metabolic disorders (e. g., obesity, diabetes) are microbiome-linked pathologies [10–12], which alter both microbial composition and functions. In addition to playing a crucial role in host metabolism, the gut microbiome also regulates immune responses [13]. For instance, gut microbiota plays a vital role in priming immune responses and microbial perturbation leads to immune dysregulation and uncontrolled inflammation [14]. Moreover, microbial metabolites such as bile acids (BAs) have been shown to modulate the functions of immune cells. Leonhardt et al. [15] reported that BAs induce monocyte dysfunction by affecting membrane-bound Takeda G-protein-coupled bile acid receptor 1 (GPBAR1/TGR5) expression. Wang et al. [16] reported that BAs skew macrophage polarization and contribute to colonic inflammation. Although excessive lipolysis leads to altered metabolism and immune regulation, the role of the gut microbiome and microbial metabolites including bile acids during excessive lipolysis is not well studied in dairy cattle.

We hypothesized that excessive adipose tissue lipolysis develops with an altered gut microbiome (composition and function) in relation to changes in immune regulation and altered host metabolism. Therefore, this study aimed to profile the gut microbiome, metabolome, and immune cell transcriptome using multi-omics tools (16S amplicon sequencing, metagenomics sequencing, targeted metabolomics, and scRNA-seq) to discover mechanisms behind excessive lipolysis-associated immunosuppression. Integration of data generated from multi-omics tools expands our knowledge behind complex processes that occur during excessive lipolysis in transition cows.

## Results

### Excessive lipolysis affected the postpartum physiological status of transition cows

The physiological status of transition cows was evaluated by profiling plasma metabolic parameters, biomarkers of inflammatory, and oxidative stress as well as by measuring milk production and body condition scores (BCS) (Table 1). There were no significant differences in any of these parameters between low/normal lipolysis (LNF) and high/excessive lipolysis (HNF) cows a week prior to calving (Table S1). However, significant changes were observed postpartum (Table 1). The concentrations of non-esterified fatty acid (NEFA, *P* < 0.01) and β-hydroxybutyrate (BHBA, *P* < 0.01), and the concentration of aspartate aminotransferase (AST;* P* = 0.01) were significantly higher in HNF cows than those in LNF cows (Table 1). The concentrations of glucose were significantly lower (*P* < 0.01) in HNF cows than in LNF cows, whereas circulating cholesterol concentrations tended to be lower (*P* = 0.06) in HNF cows than that in LNF cows. Among cows’ reproductive performances, BCS was tended to be lower (*P* = 0.06) in HNF cows when compared to LNF cows. In addition, no significant differences were observed in inflammatory and oxidative stress biomarkers between LNF and HNF cows (Table 1).

**Table 1:** Postpartum circulating plasma parameters, systemic inflammatory responses, oxidative stress status, and phenotypic characteristics of transition cows with and without excessive lipolysis (*N* = 18)

	Lipolysis status		SEM	*P* value
	LNF (*n* = 9)	HNF (*n* = 9)		
**Phenotypic characteristics**				
Milk yield, kg/d	35.1	39.0	3.13	0.42
Parity	2.50	2.11	0.37	0.49
Body condition score	2.86	2.63	0.11	0.06
**Blood parameters**				
Alanine aminotransferase, U/L	19.2	17.6	1.22	0.40
Aspartate aminotransferase, U/L	87.6	135	9.67	0.01
Total protein, g/L	74.1	68.9	2.08	0.10
Albumin, g/L	33.1	31.7	0.95	0.31
Glucose, mmol/L	3.85	3.27	0.09	< 0.01
Blood urea nitrogen, mmol/L	4.08	4.21	0.37	0.80
Creatinine, μmol/L	85.4	98.9	5.50	0.12
Cholesterol, mmol/L	2.72	2.14	0.15	0.06
Triglyceride, mmol/L	0.11	0.13	0.01	0.43
β-hydroxybutyrate, μmol/L	566	1158	70.2	< 0.01
Nonesterified fatty acid, μmol/L	424	1157	111	< 0.01
**Inflammatory biomarkers**				
Haptoglobin, U/L	387	340	28.3	0.26
Serum amyloid A, μg/mL	41.6	36.0	2.31	0.12
**Oxidative stress biomarkers**				
Superoxide dismutase, U/mL	184	172	7.87	0.30
Total antioxidant capacity, mmol/L	0.54	0.52	0.01	0.32
Catalase, U/mL	1.09	1.12	0.04	0.67
Glutathione peroxidase, U/mL	30.7	38.6	3.10	0.10
Malondialdehyde, nmol/mL	3.76	3.13	0.71	0.55

### Excessive lipolysis suppressed immune cell functions

**Fig. 1:**
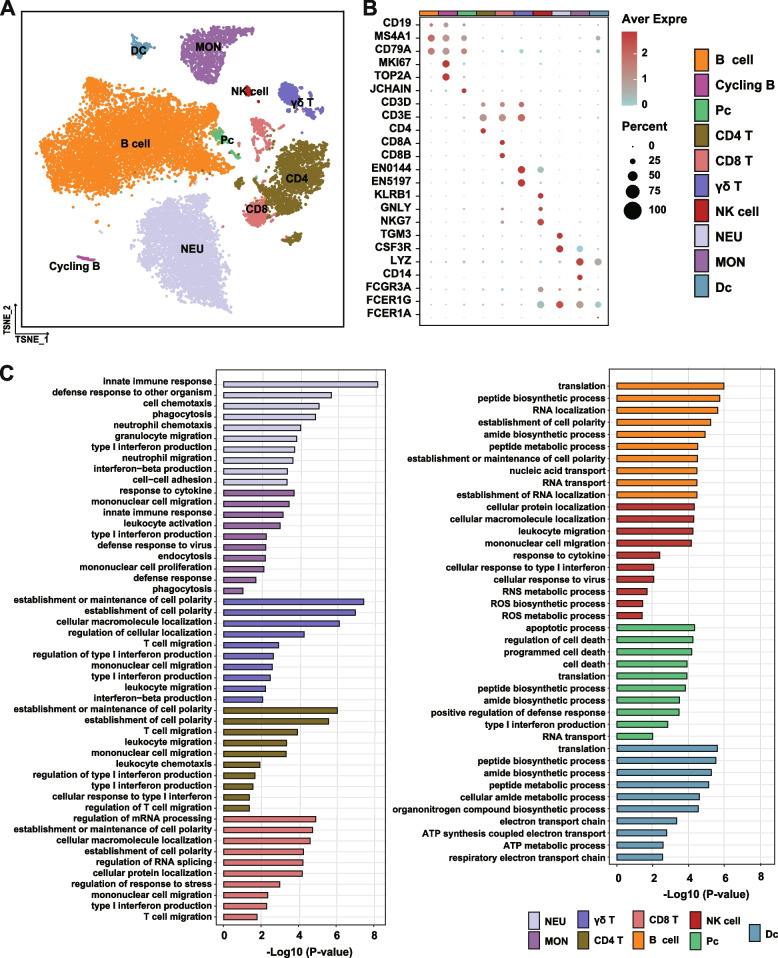
Construction of the single-cell landscape of the peripheral immune cells isolated from transition dairy cows with low (LNF) and excessive (HNF) lipolysis.** A** T-distributed stochastic neighbor embedding (T-SNE) map of major cell type clusters identified from the immune cells isolated from a LNF and a HNF transition cow. Annotation of cell types was conducted based on the highly expressed marker genes in transcript clusters. **B** Expression of marker genes in different immune cell populations. Colors represent the average expression of marker genes in each cell type and the size of dots represents % of the cells that express the genes. **C** Ten representative immune biological pathways from top 30 pathways that enriched from the downregulated differentially expressed genes of immune cells isolated from HNF cow. The pathways are presented as log10 *p* value, and the color scheme is used to indicate the immune cell population. DC dendritic cell, MON monocyte, NEU neutrophil, NK natural killer cells, PC plasma cell

In total, 26 clusters (C0-C25) were identified from 20,822 single cells based on marker genes (Table S2; Fig. S1). These clusters were then annotated into ten different major immune cell types based on the expression of well-known marker genes according to the published scRNA-seq studies of peripheral immune cells (Fig. 1A, B, Table S3). Five clusters (C0, C1, C6, C8, C12) were annotated as B cells due to the higher expression of marker genes *CD19*, *MS4A1*, and *CD79A*, while one cluster (C15) was annotated as plasma cell (PCs) due to the higher expressing *JCHAIN* (Fig. 1B). C23 was annotated as cycling B cells due to the higher expression of marker genes *CD19*, *MS4A1*, *TOP2A*, and *MKI67* (Fig. 1B). Nine clusters (C4, C7, C9, C10, C14, C16, C18, C22, and C25) were identified as T cells based on the higher expression of marker genes *CD3D* and *CD3E* (Fig. 1B). Among these T cell clusters, C4, C7, and C16 had a higher expression of CD4^+^T cell marker gene (*CD4*), C9, C14, C22, and C25 had a higher expression of CD8^+^T cell marker genes (*CD8A* and *CD8B*), while C10 and C18 had a higher expression of γδ^+^T cell marker genes (*ENSBTAG00000055197*: *TRDC* and *ENSBTAG00000000144*: *TRGC*). C20 was annotated as NK cells due to the higher expression of marker genes *KLRB1*, *GNLY*, and *NKG7*. C2, C3, and C11 were annotated as NEU due to the higher expression of marker genes *TGM3* and *CSF3R* (Fig. 1B). Among the clusters that were annotated to monocytes (MON), we identified two different subsets of monocytes (C5, C19). The higher expression of marker genes *LYZ* and CD14 in the C5 cluster suggested it contained CD14^+^MON cells, whereas the higher expression of marker genes *FCGR3A* and *LYZ* in the C19 cluster indicated it contained FCGR3A^+^MON cells (Fig. 1B). The higher expression of marker genes *LYZ*, *FCER1G*, and *FCER1A* showed the presences of dendritic cells (DCs; Fig. 1B) in C14. However, C17, C21, and C24 contained a mixture of marker genes from various immune cells and were removed from the downstream analysis.

Next, we detected the differential expressed genes (DEGs) of each cell type between LNF and HNF cows and conducted the GO enrichment analysis to identify the immune functional alteration during excessive lipolysis. Compared with LNF cows, the downregulated DEGs were mainly enriched in the biological pathways of immune cellular functions. Specifically, in NEU, the downregulated DEGs were related to neutrophil chemotaxis, neutrophil migration (*P* < 0.001), and phagocytosis (*P* < 0.001). Similarly, the downregulated DEGs in MON were related to cell migration (*P* < 0.001), proliferation (*P* = 0.006), and phagocytosis (*P* = 0.046) (Fig. 1C). Functional enrichment of downregulated genes in T cells revealed that they involved in T cell migration (*P* < 0.001) and type1 interferon production (*P* < 0.001). The downregulated DEGs in B cells, PCs, and DCs were mainly related to translation and peptide biosynthetic process (*P* < 0.001). In NK cells, functions of the downregulated DEGs were related to reactive nitrogen species (RNS; *P* = 0.020) and reactive oxygen species (ROS) metabolism (*P* = 0.039) (Fig. 1C). For the upregulated DEGs, no direct enriched biological pathways were observed in Dc, NK, and Pc cells (Fig. S2). Additionally, most of the upregulated DEGs in other cell types were enriched in the biological pathways such as translation, peptide biosynthetic process, and amide biosynthetic process that are not involved in the functions of immune cells directly (Fig. S2). Thus, we will focus on the downregulated DEGs and enriched biological pathways in HNF for the subsequent sections unless specified otherwise.

### Altered fecal bacterial community in transition cows during excessive lipolysis

**Fig. 2:**
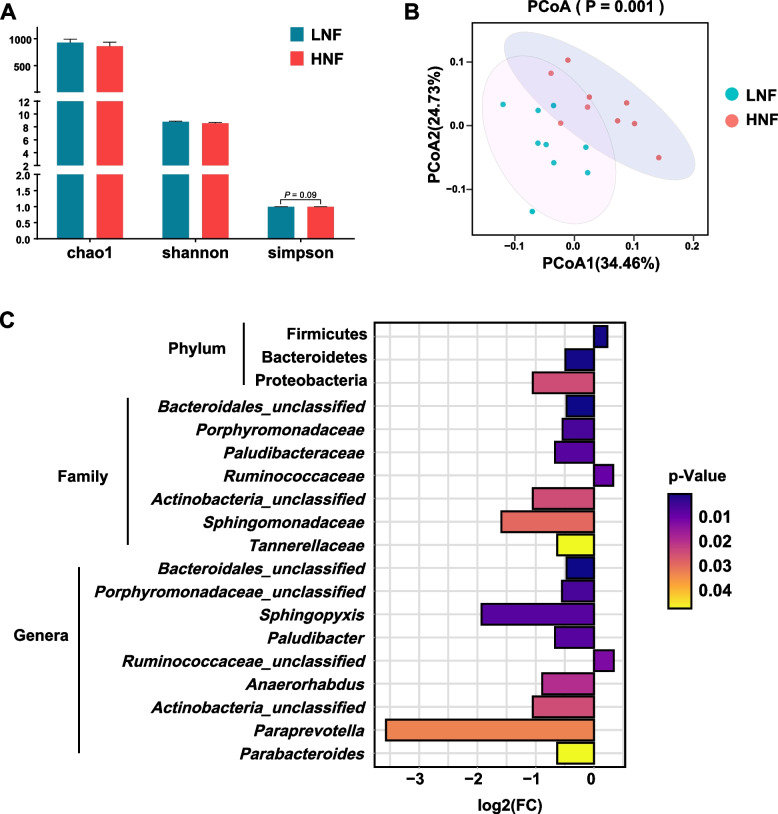
Fecal bacterial communities of transition cows (post-partum) profiled using 16S amplicon sequencing. **A** Alpha diversity of LNF and HNF cows measured using Chao1, Shannon, and Simpson indices. **B** Comparison of bacterial communities generated from LNF and HNF cows. Principle coordinate analysis was performed using Bray–Curtis distance matrices. **C** Significantly different bacterial taxa tested by wilcox.test

The use of DADA2 generated a total of 437 ASVs in all the samples (Table S4). Alpha diversity analysis revealed no significant differences in Chao1 and Shannon index between the two lipolysis groups (*P* > 0.05); however, the Simpson index (a measure of common or dominant species) tended to be lower (*P* = 0.09) in HNF when compared to LNF (Fig. 2A). Beta diversity analysis revealed a clustering of fecal bacterial communities based on lipolysis grouping (Fig. 2B).

The use of wilcox.test identified differentially abundant bacterial taxa between HNF and LNF (Fig. 2C; Table S5). At the phylum level, the relative abundance of Firmicutes (*P* < 0.001) was higher in HNF than in LNF. In contrast, the relative abundance of Bacteroidetes (*P* < 0.001) and Proteobacteria (*P* = 0.02) was lower in HNF than in LNF. Seven significantly different family taxa were observed between LNF and HNF cows. In specific, six taxa including Bacteroidales_unclassified (*P* < 0.001), Porphyromonadaceae (*P* = 0.005), Paludibacteraceae (*P* = 0.007), Actinobacteria_unclassified (*P* = 0.024), Sphingomonadaceae (*P* = 0.029), and Tannerellaceae (*P* = 0.047) with higher abundance in the LNF cows, and only Ruminococcaceae (*P* = 0.009) showed higher abundance in the HNF cows. At the genus level, the relative abundance of an unclassified *Ruminococcaceae* was higher (*P* = 0.011) in HNF than in LNF. The relative abundance of *Paraprevotella* (*P* = 0.032), *Parabacteroides* (*P* = 0.046), *Sphingopyxis* (*P* = 0.007), *Paludibacter* (*P* = 0.007), *Anaeroorhabdus* (*P* = 0.019), an unclassified *Bacteroidales* (*P* = 0.024), *Actinobacteria* (*P* = 0.024), and *Porphyromonadaceae* (*P* = 0.005) was significantly lower in HNF than LNF, among which, the *Paraprevotella* showed the biggest fold change (Log_2_FC = -3.57).

### Lipolysis status altered fecal metagenome of the transition cows

**Fig. 3:**
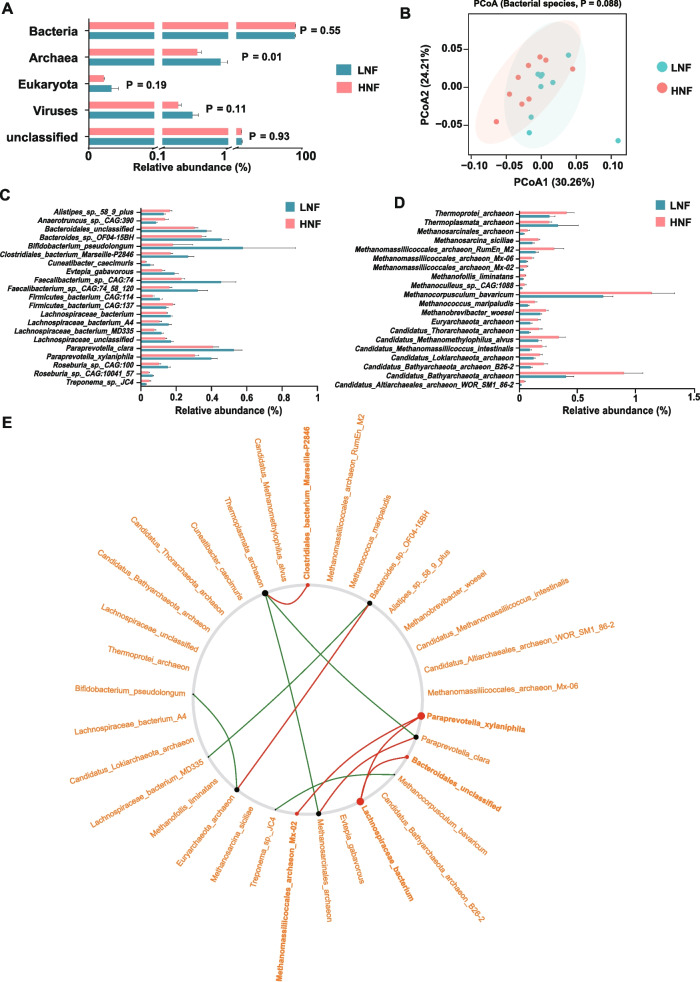
Fecal microbiomes of transition cows with varying lipolysis status profiled using metagenomic sequencing.** A** Comparison of microbial domains between LNF and HNF cows. Significantly different domains were tested by Wilcoxon rank-sum. Data is presented as mean ± SEM. **B** Bacterial community profiles of LNF and HNF fecal samples at species level visualized using principal-coordinate analysis and Bray–Curtis distance matrix. **C** Abundance of significantly different bacterial species between LNF and HNF. Bar plots represent mean ± SEM. **D** Abundance of significantly different archaea species between LNF and HNF. Bar plots represent mean ± SEM. **E** Changes between the two co-occurrence networks corresponding to LNF and HNF captured using NetShift which reflected.by the neighbor shift (NESH) cores. Node size shows the predicated “driver” scores; the big, red nodes are particularly important drivers. Edge connections in green are presented only in LNF cows, red is only in HNF cows

Taxonomic analysis of assembled metagenomics sequences revealed that bacteria (89.9% ± 0.43) dominated the fecal microbiome, followed by archaea (0.74% ± 0.07) and viruses (0.41% ± 0.05) (Fig. 3A). At the domain level, the relative abundance of archaea was significantly lower (*P* = 0.01) in HNF than in LNF. A comparison of the relative abundance of microbial taxa revealed 21 differentially abundant bacterial species between LNF and HNF (Fig. 3C). Among the differentially abundant 21 bacterial species, the relative abundance of *Alistipes sp.58 9 plus* (LNF- 0.12%; HNF-0.16%),* Anaerotruncus sp.CAG:390* (LNF-0.07%; HNF-0.14%),* Firmicutes bacterium CAG:137* (LNF-0.14%; HNF-0.18%), and *Treponema sp. JC4* (LNF-0.03%; HNF- 0.05%) was higher in HNF, while the rest (17 bacterial species) was lower in HNF when compared to LNF (Fig. 3C). There were 20 differentially abundant archaeal species between LNF and HNF. Only *Thermoplasmata archaeon* (LNF-0.03%; HNF-0.07%) was highly abundant in HNF when compared to LNF (Fig. 3D). Netshift analysis revealed a total of 14 driver species that contributed to the lipolysis-linked microbiome (Fig. 3E). Among these driver species, *Paraprevotella xylaniphila* (NESH = 0.188), *Lachnospiraceae bacterium* (NESH = 0.188), *Methanomassiliicoccales archaeon Mx-02* (NESH = 0.094), and *Clostridiales bacterium Marseille-P2846* (NESH = 0.094) had higher NESH scores and stronger betweenness (Fig. 3E and Table S6), indicating that they were the main drivers of microbiome changes linked to lipolysis status.

**Fig. 4:**
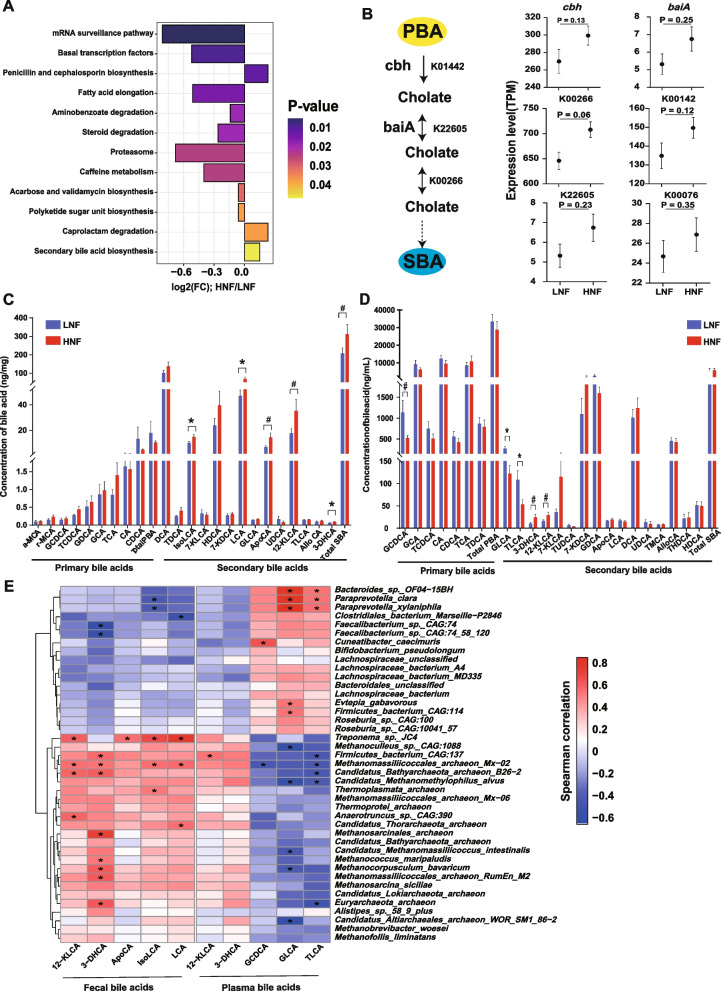
Differentially abundant KEGG pathways of fecal microbiomes of transition cows with varying lipolysis status.** A** Significantly different KEGG pathways of gut microbiome between LNF and HNF cows. **B** Simplified flow chart of the process of secondary bile acid synthesis (left) and the comparison of the abundance of major genes and entry pathways involved in the process. The *P* value was calculated using the Wilcoxon rank-sum test. PBA primary bile acid, SBA secondary bile acid. **C** Comparison of the concentration of fecal bile acids between LNF and HNF cows. **D** Concentration of plasma bile acids in LNF and HNF cows. **E** Spearman correlations between significantly different microbial species and significantly different fecal and bile acids between LNF and HNF cows. ^*^P < 0.05, ^#^0.05 < *P* < 0.1. TDCA taurodeoxycholic acid, TCA taurocholic acid, CDCA chenodeoxycholic acid, CA cholic acid, TCDCA taurochenodeoxycholic acid, GCA glycocholic acid, GCDCA glycochenodeoxycholic acid, HDCA hyodeoxycholic acid, THDCA taurohyodeoxycholic acid, AlloCA allocholic acid, UDCA ursodeoxycholic acid, DCA deoxycholic acid, LCA lithocholic acid, ApoCA apocholic acid, GDCA glycodeoxycholic acid, 7-KDCA, 7-ketodeoxycholic acid, 7-KLCA 7-ketolithocholic acid, 12-KLCA 12-ketolithocholic acid, 3-DHCA 3-dehydrocholic acid, TLCA taurolithocholic acid, GLCA glycolithocholic acid, total α-MCA α-Muricholic acid, γ-MCA γ-muricholic acid, isoLCA isolithocholic acid, TMCA tauro-muricholic acid, TUDCA tauroursodeoxycholic acid, TPBA total primary bile acid, TSBA total secondary bile acids

Assignment of microbial functions using KEGG revealed a total of 169 KEGG pathways in the fecal metagenomes of transition cows (Table S7). Among these KEGG pathways, the abundance of 12 pathways was significantly different (*P* < 0.05) between LNF and HNF (Fig. 4A). The relative abundance of pathways related to “lipid metabolism” including “fatty acid elongation” (*P* = 0.015) and “steroid degradation” (*P* = 0.019) was lower in HNF than LNF. Besides, the abundance of gut microbial secondary bile acid (SBA) biosynthesis pathways was significantly higher in HNF cows than in LNF cows (*P* = 0.047). However, no significant differences were observed in the major genes *cbh* (*P* = 0.13) and *baiA* (*P* = 0.25) that were involved in the SBA synthesis process (Fig. 4B). Similarly, the abundance of major enzymes that are involved in SBA syntheses such as acyl-CoA synthetase (K00142) and 3alpha-hydroxycholanate dehydrogenase (K22605) was not significantly different between HNF and LNF. In contrast, 7-alpha-hydroxysteroid dehydrogenase (K00076) tended to be higher (*P* = 0.06) in HNF cows when compared to LNF (Fig. 4B).

### Altered bile acid profiles in transition cows with excessive lipolysis

Targeted metabolomics analysis of fecal bile acids (BAs) revealed that total secondary BAs tended to be higher (*P* = 0.10) in HNF than in LNF. Moreover, the concentration of lithocholic acid (LCA, *P* = 0.04), isolithocholic acid (IsoLCA, *P* = 0.04), and 3-dehydrocholic acid (3-DHCA, *P* = 0.04) was significantly higher in HNF when comparing to LNF. The concentrations of 12-ketolithocholic acid (12-KLCA, *P* = 0.09) and apocholic acid (ApoCA, *P* = 0.06) tended to be higher in the HNF than in the LNF (Fig. 4C). The proportions of 12-KLCA (*P* = 0.06) and ApoCA (*P* = 0.07) also tended to be higher in HNF than LNF (Fig. S3A).

Among plasma BAs, the concentrations of glycolithocholic acid (GLCA, *P* = 0.02) and taurolithocholic acid (TLCA, *P* = 0.03) were lower in HNF than in LNF. Moreover, the concentrations of glycochenodeoxycholic acid (GCDCA) tended to be lower (*P* = 0.06) in HNF than in LNF. In contrast, the concentrations of 3-DHCA (*P* = 0.08) and 12-KLCA (*P* = 0.10) tended to be higher in HNF than in LNF (Fig. 4D). The proportion of GCDCA was lower (*P* = 0.04) in HNF than in LNF, while the proportion of the 7-ketodeoxycholic acid (7-KDCA, *P* = 0.09) and 12-KLCA (*P* = 0.07) tended to be higher in HNF than LNF (Fig. S3B). The proportion of 3-DHCA (*P* = 0.04) was higher in HNF than in LNF.

The use of a correlation analysis revealed that the relative abundance of *Treponema sp. JC4* and *Methanomassiliicoccales archaeon Mx02* in feces was positively correlated with the concentrations of fecal BAs including 12-KLCA, 3-DHCA, ApoCA, IsoLCA, and LCA (*P* < 0.05). The relative abundances of *Bacteroides sp. OF04−15BH*, *Paraprevotella clara*, and *Paraprevotella xylaniphila* were positively correlated with the concentrations of GLCA and TLCA in plasma (Fig. 4E, Table S8).

### Suppression of monocyte functions in HNF cow was linked to bile acid metabolism

**Fig. 5:**
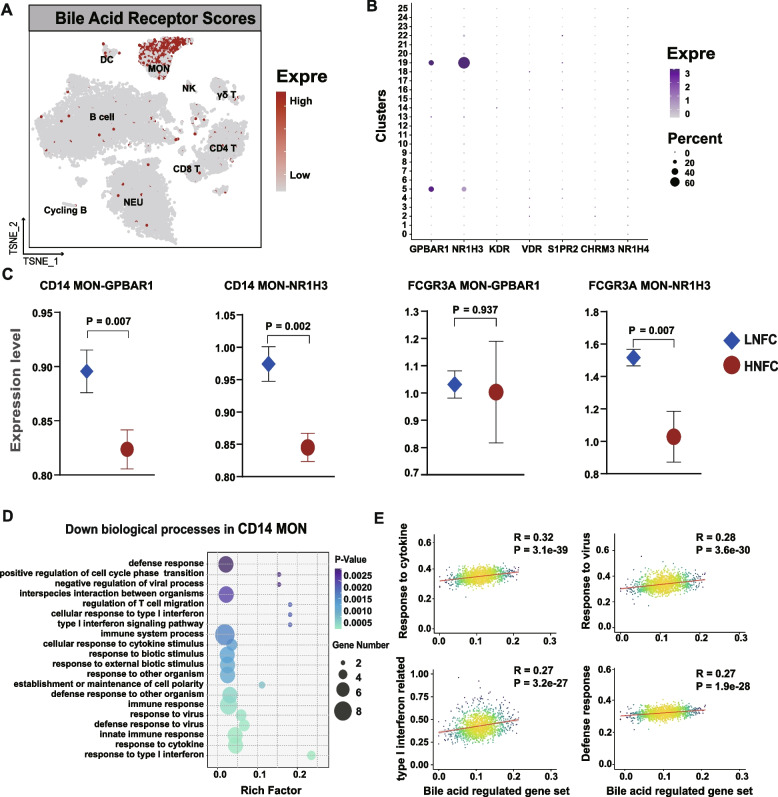
Association between bile acids and immune cell functions.** A** T-distributed stochastic neighbor embedding (T-SNE) plot of expressing scores of bile acid receptors (BARs) in immune cells. Red spots represent cells that express BARs. **B** Expression of bile acid receptor genes in each cell cluster. The color represents the expression level of BARs, and the size of the dots represents the percentage of the cells that express the genes. **C** The comparison of *GPBAR1* and *NR1H3* expression in CD14^+^MON and FCGR3A^+^MON cells isolated from a LNF cow and a HNF cow. The analysis included a total of 1568 CD14^+^MON and FCGR3A^+^MON from both cows. **D** Top 20 enriched biological processes from downregulated DEGs of CD14^+^MON cells isolated from HNF cow compared to LFN cow. **E** Association between the expression of genes related to bile acid metabolism and down-regulated functions of CD14^+^MON

Bile acid receptors are one of the interfaces between the gut microbiota and host immune regulation. Therefore, we evaluated the link between the expression of bile acid receptor genes (BARs) and immune cell functions. We identified seven BARs (*GPBAR1*,* KDR*,* VDR*,* SIPR2*,* CHRM3*,* NR1H4*, and *NR1H3*) in the single-cell transcriptome data generated from the immune cells isolated from cows with varying lipolysis status. The results showed that MON exhibited a uniquely higher BAR expressing score compared with other cell types (Fig. 5A). In specific, the higher expression of *GPBAR1* and *NR1H3* was observed in MON (Fig. 5B). For both two BARs, the expression of *GPBAR1* was lower (*P* = 0.007) only in CD14^+^MON, while the expression of *NR1H3* was lower in both CD14^+^MON (*P* = 0.002) and FCGR3A^+^MON (*P* = 0.007) isolated from the HNF cow than those in LNF cow (Fig. 5C). Monocytes were selected to further understand the links between the gut microbiome and immune regulation during excessive lipolysis. Enrichment of GO function of the DEGs (Table S9) expressed in CD14^+^MON and FCGR3A^+^MON revealed that immune pathways related to “immune response,” “response to cytokine,” “response to type 1 interferon,” and “defense response to virus” were significantly downregulated in CD14^+^MON isolated from HNF cow (Fig. 5D). Moreover, “cell migration” and “phagocytosis” were decreased in FCGR3A^+^MON isolated from HNF (Fig. S4A). Integrated analysis revealed that downregulated DEGs related to BA regulation (Table S10) and four enriched immune functions (response to cytokine, response to virus, defense response, and type1 interferon) of monocytes were only positively correlated in CD14^+^MON but not in FCGR3A^+^MON (Fig. 5E and Fig. S4B).

## Discussion

Perturbed metabolism and immunosuppression are signature outcomes that develop in parallel with excessive lipolysis in transition cows [17]. Mobilization of body fat is a vital biological response to compensate for energy deficiency due to the negative energy balance during the transition period in dairy cows [1]. Therefore, a low to mild lipolysis is expected in transition cows [18]. Despite the extensive literature on altered metabolism and immunosuppression during excessive lipolysis of transition cows, there is a lack of understanding on the mechanisms behind these outcomes. In addition, metabolic disorders have been linked to microbiome perturbation in humans and mice [10–12]. Here, we report that lipolysis status (HNF vs. LNF) is related to fecal microbial composition, microbial functions linked to SBA synthesis, host BA profiles, and monocyte functions in transition cows using multi-omics approaches. This is the first study to reveal relationships between lipolysis status-dependent changes and fecal microbiota community and functions of transition cows, which suggests that excessive lipolysis could be a microbiome-linked pathology. Profiling of the microbiome (using both 16S amplicon sequencing and metagenomics sequencing) of transition cows revealed microbial perturbations (compositional and functional), and BA profile (using targeted metabolomics) in plasma and feces showed BA metabolism disorder contribute to the immunosuppression of peripheral MON (using scRNA-seq) in the transition cows with excessive lipolysis.

As a signaling molecule, increasing studies reported that BAs play important roles in the metabolism and immunity of human beings [19] or animals [20]; however, limited information was observed in dairy cows. Here, we found significant changes in BA profile in transition cows with excessive lipolysis. Although there are few studies that focus on BA metabolism in perinatal dairy cows, some clues about BA disorders from the experiments in dairy cows during the transition period can be dug out. For example, McCabe et al. [21] reported that BA biosynthesis and secretion were decreased based on the liver transcriptomic analysis in the transition cows with severe NEB (higher NEFA state). In the results of the liver transcriptome that reported by Gao et al. [22], it is found that BA biosynthesis and secretion were downregulated from dairy cattle from prepartum to postpartum. These clues imply that the BA metabolism may be perturbed in the transition dairy cows especially in those cows with higher lipolysis. However, the abovementioned results can only support part of BA metabolism changes, specifically the primary BA synthesis in the liver. Our results suggest that SBA which is formed by gut microbiota metabolizing primary BAs [23] is also significantly altered, for example, the decreased plasma LCA and DCA concentrations in the transition cows with excessive lipolysis. Therefore, it is natural to speculate that this may be attributed to the alterations in the composition and functions of the gut microbiome. The consistent results of 16S amplicon and metagenomics sequencing confirmed the assumption. As we know, most of the microbial studies are predominantly biased toward the rumen microbiome [24–26]; recently, it suggested that gut microbiome plays vital roles in host metabolism and animal health through building a comprehensive gastrointestinal microbial gene catalog in dairy cows [27]; our results supported this viewpoint and extended the understanding of gut microbiome in dairy cows. Additionally, previous studies were mainly focused on the effects of metabolic disorders such as fatty acids [5, 6], glucose [28], and hormones [29] in the perinatal dairy cows. Here, we provide a new important clue for the exploration of the behind mechanisms of disorders occurred in transition cows with excessive lipolysis, namely, we should pay more attention to the alteration of gut microbial and BA metabolism as well as its effects on the transition dairy cows with excessive lipolysis.

Many studies have shown that BAs contribute to the functions of immune cells through binding to specific BA receptors (BARs) [30, 31]. Interestingly, we found that the MONs showed uniquely higher BARs scores that responded to BA, indicating the BA could regulate the functions of MON rather than other cell types. Moreover, the expression of BARs on MON is affected by the lipolysis status. Furthermore, the significantly higher expression of *GPBAR1*, the strong relationship between BA metabolism, and reduced functions of MON together suggested that CD14^+^MON is more sensitive to BA than FCGR3A^+^MON. *GPBAR1* (also known as *TGR5*) is a member of G-protein-coupled receptor superfamily for BA, and it is evidenced that *GPBAR1* is expressed by peripheral blood-derived monocyte/macrophages and appears to mediate the immunomodulatory actions [32]. Previous studies revealed that the activation of *GPBAR1* works in the activation, migration, and anti-inflammatory cytokine production of MON in mice and humans [33–35]. Therefore, the functions of CD14^+^MON suppression in HNF cows may be attributed to the decreased expressing *GPBAR1*. Additionally, it is thought that the GLCA and TLCA are the most preferred binding ligands of GPBAR1 [36]. Thus, the *GPBAR1* decreased expression in HNF cows might be caused by the reduction of plasma GLCA and TLCA. In sum, these results indicate that the disturbed BA homeostasis (lower peripheral GLCA and TLCA) contributed to the immunosuppression of CD14^+^MON via diminishing the expression of *GPBAR1* in transition cows with excessive lipolysis. However, future studies to explore the causation effects of SBAs on immunosuppression are necessary to validate the proposed mechanism in the present study.

## Conclusions

Lipolysis is a complex biological process that leads to immunosuppression in transition cows, which affects the health and production of the dairy industry [37]. Here, we report that the integration of multi-omics data provides a holistic approach for the researchers to undrstand complex process. To the best of our knowledge, this is the first study to combine gut microbiome, metabolome, and host single-cell immune transcriptome in dairy cattle to investigate excessive lipolysis in transition cows. We report that microbial biosynthesis of SBAs and fecal SBAs were both increased in cows with excessive lipolysis. In contrast, the functions of monocytes related to cell migration, phagocytosis, response to cytokine, and defense responses to the virus were decreased during excessive lipolysis. Therefore, we propose that the reduced concentration of conjugated BAs in plasma (GLCA, TLCA) that activates *GPBAR1* on immune cells (especially on CD14^+^MON) leads to an immunosuppression in transition cows during excessive lipolysis. Our analysis also suggested that *Bacteroides sp. OF04 − 15BH*, *Paraprevotella clara*, *Paraprevotella xylaniphila*, and *Treponema sp. JC4* might be playing an important role in the biosynthesis of SBAs, implying excessive lipolysis is microbiome-linked pathology in dairy cows. In conclusion, our study revealed potential links between the gut microbiome, BA metabolism, and immunosuppression in transition cows with excessive lipolysis.

## Methods

### Experimental design and sample collection

Holstein dairy cows (*n* = 63) without veterinary intervention were selected from a large cohort (Hangjiang Dairy Farm, Hangzhou, China) of 2000 dairy cows seven days prior to the expected calving date. All cows were raised and managed under the same conditions including diet, water, and environment. Blood samples were collected from the coccygeal vein of cows 7 days prior to expected calving date and 7 days after calving using EDTA vacutainers. Moreover, fecal samples were collected at seven days postpartum from the rectum of cows by using sterilized gloves before morning feeding, transferred into 50-mL sterile tubes, and snap-frozen in liquid nitrogen.

Cows with excessive lipolysis were identified based on the plasma concentration of NEFA on 7 days postpartum [5]. Briefly, cows with plasma NEFA concentrations > 750 μmol/L on day seven postpartum were defined as high/excessive lipolysis (HNF), whereas those with plasma NEFA concentrations < 600 μmol/L were defined as low/normal lipolysis (LNF). We selected 18 dairy cows (LNF, *n* = 9; HNF, *n* = 9) out of 63 cows based on plasma NEFA concentrations (power = 0.9) after controlling for parity, milk yield, and body condition score (BCS). Body condition scores were measured by two people using a 5-point scale (1 = thin, 5 = fat) method described by Edmonson et al. [38] at 3-time points (06:00, 14:00, 20:00).

### Plasma parameters measurement and statistical analysis

Blood samples collected from 63 cows 7 days before and after calving were used to measure plasma metabolites. First, blood samples were centrifuged at 3000 × *g* for 15 min at 4 °C to collect plasma to measure the concentrations of glucose, total protein, blood urea nitrogen, NEFA, BHBA, cholesterol, triglycerides, albumin, superoxide dismutase, creatinine, alanine aminotransferase, and AST using an AutoAnalyzer 7020 instrument (Hitachi High-Technologies Corporation, Tokyo, Japan) and commercial kits (Ningbo Medical System Biotechnology Co., Ltd., Ningbo, China). The levels of catalase, glutathione, glutathione peroxidase, malondialdehyde, haptoglobin, amyloid, and total antioxidant capacity were measured using commercial assay kits (Nanjing Jiancheng Bioengineering Institute, Nanjing, China) according to the manufacturer’s instructions.

Statistical analyses were performed using Prism (GraphPad Software Inc 8.0, La Jolla, CA, USA). Student’s *t* test between the two groups was used for comparisons. Significances were declared at *P* ≤ 0.05, and 0.05 < *P* ≤ 0.10 were considered as a significant trend.

### 16S amplicon sequencing of the fecal microbial community

The total DNA was extracted from fecal samples using the E.Z.N.A.®Stool DNA Kit (#D4015, Omega, Inc., USA). V3–V4 regions of the bacterial 16S rRNA gene were amplified using a universal bacterial primer pair (341F: 5′-CCTACGGGNGGCWGCAG-3′; 805R: 5′-GACTACHVGGGTATCTAATCC-3′) [39]. Then, purified PCR products using AMPure XT Beads (Beckman Coulter Genomics, Danvers, MA, USA) were used to prepare sequencing libraries using TruSeq Nano DNA LT Library Preparation Kit (FC-121–4001). Sequencing was performed on an Illumina NovaSeq 6000 with PE250 mode at LC-Bio Technology Co., Ltd. (Hangzhou, China).

First, raw sequences were demultiplexed into paired-end FASTQ files. Quality filtering was performed to obtain high-quality clean tags through fqtrim (v 0.94) [40] by removing the low-quality reads (quality scores < 20), short reads (< 100 bp), and reads containing more than 5% “*N*” records. DADA2 [41] with default parameters was used for denoising and generating amplicon sequence variants (ASVs) of quality reads that were de-replicated at 100% sequence identity and clustered at 99% sequence identity. Sequences were aligned using BLAST and taxonomic classification was done using SILVA (v 138) database. Microbial taxa with relative abundance > 0.01% in more than 50% of the samples were used in the downstream analysis. Bacterial community profiles were compared using alpha and beta diversity analyses (with Bray–Curtis distance) within QIIME2. Chao1, Shannon, and Simpson indices were used to calculate the alpha diversity of fecal microbial communities generated from HNF and LNF cows, and differences were compared using one-way ANOVA. Beta diversity analysis was conducted by using Bray–Curtis distance matrix and principle coordinate analysis (PCoA) [42]. The wilcx test was used to identify the differentially abundant taxa, and significances were declared at *P* < 0.05.

### Metagenomics sequencing of fecal microbiome

DNA libraries were constructed using the TruSeq Nano DNA Library Preparation Kit-Set (#FC-121–4001, Illumina, USA) following the manufacturer’s instructions. Metagenome libraries were then sequenced on an Illumina NovaSeq 6000 platform with PE150 at LC-Bio Technology Co., Ltd. (Hangzhou, China). Sequencing adapters were removed from de-multiplexed raw sequences using cutadapt (v 1.9). Then, the low-quality reads (quality scores < 20), short reads (< 100 bp), and reads containing more than 5% “*N*” records were trimmed by using the sliding-window algorithm method in fqtrim (v 0.94) [34]. Quality filtered reads were first aligned to bovine genome (bosTau8 3.7, https://doi.org/10.18129/B9. bioc. BSgenome. Btaurus. UCSC. bosTau8) by using bowtie (v 2.2) to filter out host contaminations [43]. Then, the remaining reads were subjected to de novo assembly for each sample using IDBA-UD (v 1.1.1) [44] and used to assign microbial functions and taxonomy. MetaGeneMark (v 3.26) [45] was used to predict the coding regions (CDS) of the assembled contigs, and CDS sequences of all samples were clustered using CD-HIT (v 4.6.1) to obtain unigenes. DIAMOND (v 0.9.14) was used to perform a taxonomic assessment of the gut microbiota based on the RefSeq database [46]. Microbial taxa with a relative abundance > 0.01% in more than 50% of the samples were used for downstream analysis. The wilcx test was used to identify the differentially abundant species, and significances were declared at *P* < 0.05. An assignment of microbial functions was done using the Kyoto Encyclopedia of Genes and Genomes (KEGG). The abundance of KEGG pathways was normalized to transcripts per million (TPM) [47], and pathways with > 5 TPM in at least 50% of the samples were used for downstream analysis.

NetShift [48] was used to identify microbial species serving as “drivers” of the altered microbiomes during excessive lipolysis. Briefly, microbiomes from LNF and HNF cows were defined as the control and case, respectively. Then, the betweenness value, which quantifies the importance of each selected species was obtained by a Spearman’s rank correlation analysis in LNF and HNF cows. Next, the betweenness values of each species were input into the NetShift package to calculate neighbor shift (NESH) cores. Node sizes are proportional to their scaled NESH scores, and the node is colored red if its betweenness value increases from control to case.

### Profiling of blood and fecal bile acids and the statistical analysis

Profiling of bile acids (BA) in feces was done according to a method described by Hu et al. [49]. Briefly, 30 mg of feces was homogenized in 300 μL of pre-cooled ultrapure water. Then, precooled methanol (500 μL) and internal standard (10 μL) were added into 100 μL homogenized feces and incubated at − 20 °C for 20 min after vortexing. Plasma (100 μL) was directly incubated with precooled methanol and internal standard. After centrifuging at 14,000 g for 15 min, the supernatants were collected for vacuum drying and then resuspended in 100 μL methanol to water (1:1, v/v). Analyses were performed using an UHPLC (Waters Ltd.) coupled online to 5500 QTRAP Mass Spectrometry (AB SCIEX, USA). The peak area and retention time were generated by Multiquant software. The internal standards of BAs were used to correct the retention time and to identify metabolites.

Statistical analysis was performed using Prism (GraphPad Software Inc 8.0, La Jolla, CA, USA). The Student’s *t* test between the two groups was used for comparisons. Significances were declared at *P* ≤ 0.05 and 0.05 < *P* ≤ 0.10 were considered as significant trends. Spearman’s rank correlation analysis (R packages Hmisc v 4.6.0) was used to determine the associations between differentially abundant microbial species (from metagenomics sequencing) and BA metabolites. *P* values were generated using the *t* or *F* distributions, and *P* < 0.05 was regarded as significantly correlation.

### Single-cell RNA sequencing (scRNA-seq) of peripheral blood immune cells and data processing

One cow was randomly selected from LNF and HNF to isolate peripheral blood mononuclear cells (PBMCs) and granulocytes to perform scRNA-seq analysis. Detailed methods on isolation of immune cells and performing scRNA-seq have been previously published [50]. In brief, 1 mL of the blood was mixed with 1 mL of PBS and then slowly poured onto 2 mL density separation fluid (Histopaque®-1077, SIGMA, RNBJ0579). Then, all samples were centrifuged at 700 × *g* for 20 min at 20℃. The buffy coat containing PBMCs and the bottom layer containing granulocytes were transferred into new tubes containing 3 mL PBS and centrifuged at 700 × *g* for 20 min at 20℃. Cells were collected and resuspended in 400 µL PBS and 10 mL lysate (Miltenyi Biotec, 130–094-183). Samples were centrifuged again at 100 × *g* for 7 min at 12 °C following incubating on ice for 10 min. After washing with PBS, PBMCs and granulocytes were collected with 1 mL of PBS and stored on ice until subsequent analysis. PBS used in every isolation step contained 0.1% bovine serum albumin. Dead cells and cellular debris were removed using the MACS Dead Cell Removal Kit (Miltenyi Biotec, Bergisch Gladbach, Germany) following the manufacturer’s instructions. Then, the total number of cells was counted using a Countess II Automated Cell Counter (ThermoFisher, USA). The viability of isolated cells was checked (over 95%) using trypan blue.

Cell suspensions were diluted to a concentration of 700 to 1200 cells/μL with 1 × PBS containing 0.04% BSA prior to 10X Genomics sequencing. A high-quality single-cell suspension was loaded in a 10X Genomics Chromium machine to capture cells and construct cDNA libraries according to Single-Cell 3′ Protocol recommended by the manufacturer. RNA-seq was performed using the NovaSeq 6000 system (PE150) at LC-Bio Technology Co., Ltd. (Hangzhou, China).

Raw reads were demultiplexed and converted into FASTQ format using Illumina bcl2fastq software (v 2.20). Sample demultiplexing, barcode processing, and single-cell 3′ gene counting were performed using CellRanger (version 3.1.0). Following quality filtering, scRNA-seq reads were aligned to ARS-UCD1.2 cattle reference genome (ftp.ensembl.org/pub/release-99/fasta/bos_taurus). The output of CellRanger was loaded into Seurat (v 4.0.3) for cell filtration, dimensional reduction, clustering, differential gene expression analysis, and marker gene screening of scRNA-seq data. Overall, cells with 500 to 4000 genes, UMI counts less than 50,000, and a mitochondrial gene ratio smaller than 15% were retained for downstream analysis. The DoubletFinder package (v 2.0.3) was used to remove doublets [51]. We further reduced the dimensionality of the variable genes in all high-quality cells using Seurat (v 4.0.3) and applied the t-distributed stochastic neighbor embedding (T-SNE) algorithm to project the variables into a two-dimensional space. The batch correction was performed using Harmony [52] (v 0.1.0) and “FindAllMarkers” function was used to identify the marker genes (| ‘avg_logFC’|> 0.25 and ‘*P*_val_adj’ < 0.05) of each cluster. The annotate of each cell type was based on the published well-known marker genes that were reported in the scRNA-seq studies of peripheral immune cells [53–55]. Based on the gene × cells matrix, differentially expressed genes between LNF and HNF cows of each cluster were identified using a Wilcoxon rank sum test within the “FindAllMarkers” function [50]. Genes with FDR adjusted *P* value of less than 0.05, log fold change greater than 0.5 or less than -0.5, and average expression counts of more than 15% were regarded as differentially expressed genes (DEGs). The functional enrichment analysis of the DEGs was performed sing “enrichGO” in the clusterProfiler R package [56] based on the dataset “org.Bt.eg.db.”

### Gene set scoring analysis

The “AddModuleScore” function of the Seurat R package (v 4.0.3) was used to compute the signature score of the gene set including the bile acid receptors gene set in all immune cells, as well as the downregulated genes related to BA regulation, response to cytokine, response to virus, defense response, and type1 interferon related in monocytes. The impact of lipolysis status on signature scores of immune cells was analyzed using a Wilcoxon rank sum test in R (v 4.1.0). Additionally, the correlation analysis between these pathways in monocyte was based on these scores [57].

## Supplementary Information

**Additional file 1: Table S1.** Comparison of plasma physiological parameters, inflammation, oxidative stress and phenotypic characteristics between cows with and without excessive lipolysis at -7d before calving. **Table S2.** The marker gene list of the 26 immune cell clusters. **Table S3.** The specific markers of major cell types. **Table S4.** Identified high-quality ASVs in LNF and HNF cows. **Table S5.** The significantly defferent microbes between LNF and HNF cows by wilcox.test. **Table S6.** The results of Netshift analysis from significant microbial species between LNF and HNF cows. **Table S7.** The enriched KEGG pathways (level3) in the gut of LNF cows and HNF cows. **Table S8.** Correlations between significant different species and fecal and plasma bile aicds. **Table S9.** The differential down-regulated genes between LNFC and HNFC in CD14+MON and FCGR3A+MON. **Table S10.** Bile acid regulated gene list.

**Additional file 2: Figure S1.** The single-cell landscape of the peripheral immune cells in cows with low (LNF) and high lipolysis (HNF). A. T-distributed stochastic neighbor embedding (T-SNE) plot map of cell type clustering from the peripheral immune cells of LNF and HNF cows. B. The violin plot of the marker genes expressed in each clusters. **Figure S2.** Top ten representative immune biological pathways that enriched from the up-regulated differential expressed genes of immune cells isolated from HNF cow. Pathways are presented as log10 *p*-value and color scheme is used to indicate immune cell population. MON: monocyte; NEU: neutrophil. **Figure S3.** The bile acid profile in plasma and feces of cows with low (LNF) and high lipolysis (HNF). A. The percentage of plasma bile acid in two groups. B. The percentage of fecal bile acid in two groups. TDCA: Taurodeoxycholic acid; TCA: Taurocholic acid; CDCA: Chenodeoxycholic acid; CA: Cholic acid; TCDCA: Taurochenodeoxycholic acid; GCA: Glycocholic acid; GCDCA: Glycochenodeoxycholic acid; HDCA: Hyodeoxycholic acid; THDCA: Taurohyodeoxycholic acid; AlloCA: Allocholic acid; UDCA: Ursodeoxycholic acid; DCA: Deoxycholic acid; LCA: Lithocholic acid; ApoCA: Apocholic acid; GDCA: Glycodeoxycholic acid; 7-KDCA: 7-ketodeoxycholic acid; 7-KLCA: 7-Ketolithocholic acid; 12-KLCA: 12-ketolithocholic acid; 3-DHCA: 3-dehydrocholic acid; TLCA: Taurolithocholic acid; GLCA: Glycolithocholic acid; Total α-MCA: α-Muricholic acid; γ-MCA: γ-muricholic acid; isoLCA: Isolithocholic acid; TMCA: Tauro-muricholic acid; TUDCA: Tauroursodeoxycholic acid. LNF: cows with low lipolysis; HNF: cows with high lipolysis. * *P*-value < 0.05; # 0.05 < *P*-value < 0.10. **Figure S4.** The functional changes and associations with bile acid related gene set. A. The enriched decreased biological process of FCGR3A^+^MON in HFNC compared to LFNC. B. Correlation of bile acid metabolism to the major decreased functions in FCGR3A^+^MON. LNFC: cow with low lipolysis; HNFC: cow with excessive lipolysis; MON: monocyte.

## Data Availability

The 16S rRNA gene sequences and metagenome sequences of 18 cattle fecal samples were deposited into the National Microbiology Data Center (NMDC; https://nmdc.cn/) under the accession numbers: NMDC10018108. The data of scRNA-seq was updated to the China National Center for Bioinformation (CNCB; https://www.cncb.ac.cn/?lang=en) with accession number: PRJCA009169. The supplementary tables can be access to and downloaded from Figshare via the link: https://figshare.com/s/ccd55b0d25d0e4f6cd0e.
