# Supplementary material for: Gut microbiome is linked to functions of peripheral immune cells in transition cows during excessive lipolysis
Source: Microbiome. 2023 Mar 3;11:40. doi: 10.1186/s40168-023-01492-3 (PMC9983187; doi:10.1186/s40168-023-01492-3)
Supplement: Supplementary file 3 — Additional file 2: Figure S1. The single-cell landscape of the peripheral immune cells in cows with low (LNF) and high lipolysis (HNF). A. T-distributed stochastic neighbor embedding (T-SNE) plot map of cell type clustering from the peripheral immune cells of LNF and HNF cows. B. The violin plot of the marker genes expressed in each clusters. Figure S2. Top ten representative immune biological pathways that enriched from the up-regulated differential expressed genes of immune cells isolated from HNF cow. Pathways are presented as log10 p-value and color scheme is used to indicate immune cell population. MON: monocyte; NEU: neutrophil. Figure S3. The bile acid profile in plasma and feces of cows with low (LNF) and high lipolysis (HNF). A. The percentage of plasma bile acid in two groups. B. The percentage of fecal bile acid in two groups. TDCA: Taurodeoxycholic acid; TCA: Taurocholic acid; CDCA: Chenodeoxycholic acid; CA: Cholic acid; TCDCA: Taurochenodeoxycholic acid; GCA: Glycocholic acid; GCDCA: Glycochenodeoxycholic acid; HDCA: Hyodeoxycholic acid; THDCA: Taurohyodeoxycholic acid; AlloCA: Allocholic acid; UDCA: Ursodeoxycholic acid; DCA: Deoxycholic acid; LCA: Lithocholic acid; ApoCA: Apocholic acid; GDCA: Glycodeoxycholic acid; 7-KDCA: 7-ketodeoxycholic acid; 7-KLCA: 7-Ketolithocholic acid; 12-KLCA: 12-ketolithocholic acid; 3-DHCA: 3-dehydrocholic acid; TLCA: Taurolithocholic acid; GLCA: Glycolithocholic acid; Total α-MCA: α-Muricholic acid; γ-MCA: γ-muricholic acid; isoLCA: Isolithocholic acid; TMCA: Tauro-muricholic acid; TUDCA: Tauroursodeoxycholic acid. LNF: cows with low lipolysis; HNF: cows with high lipolysis. * P-value < 0.05; # 0.05 < P-value < 0.10. Figure S4. The functional changes and associations with bile acid related gene set. A. The enriched decreased biological process of FCGR3A+MON in HFNC compared to LFNC. B. Correlation of bile acid metabolism to the major decreased functions in FCGR3A+MON. LNFC: cow with low lipolysis; HNFC: cow with excessi [file 40168_2023_1492_MOESM2_ESM.docx]

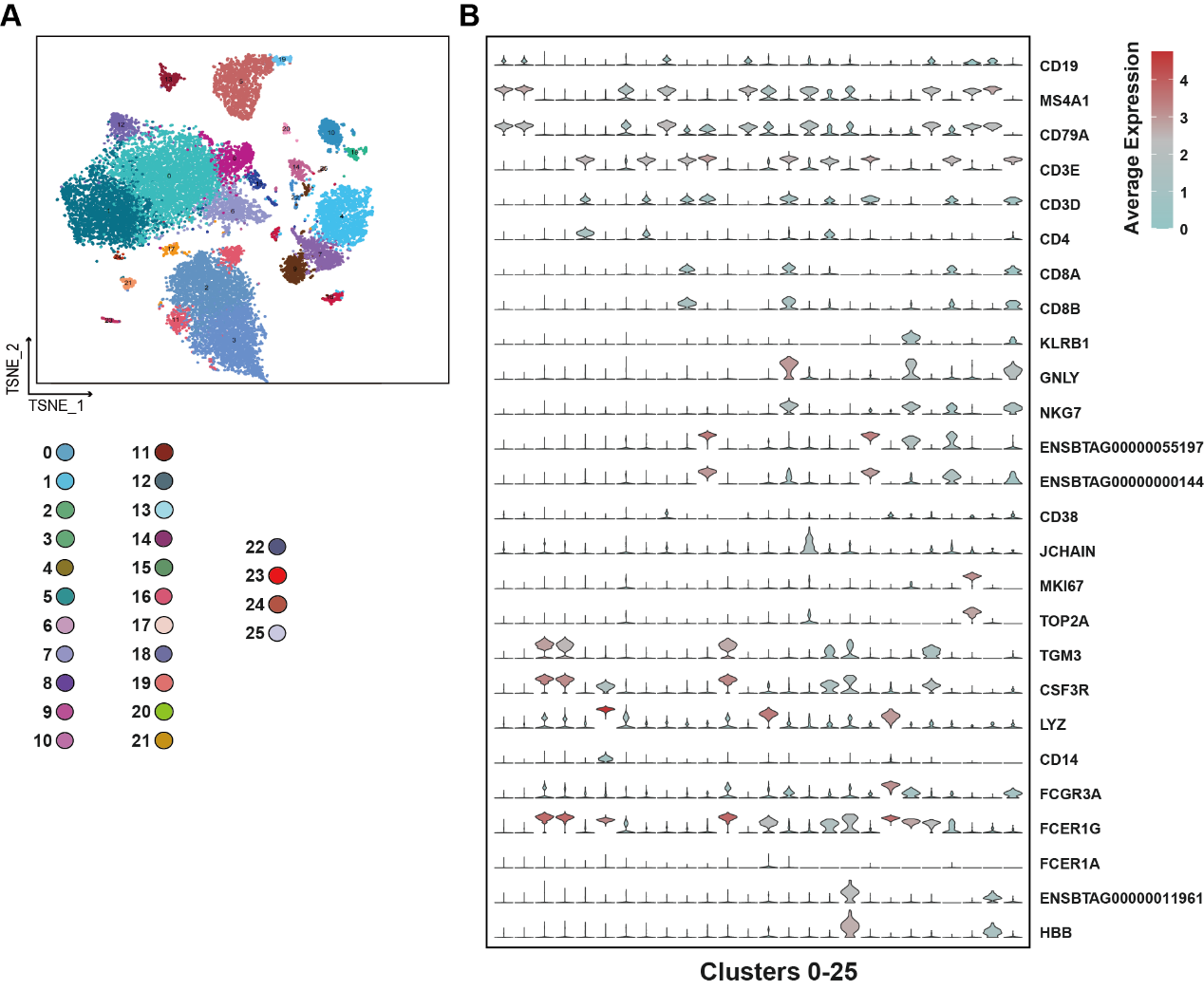


**Figure S1.** **The single-cell landscape of the peripheral immune cells in cows with low (LNF) and high lipolysis (HNF). A.** T-distributed stochastic neighbor embedding (T-SNE) plot map of cell type clustering from the peripheral immune cells of LNF and HNF cows. **B.** The violin plot of the marker genes expressed in each clusters.


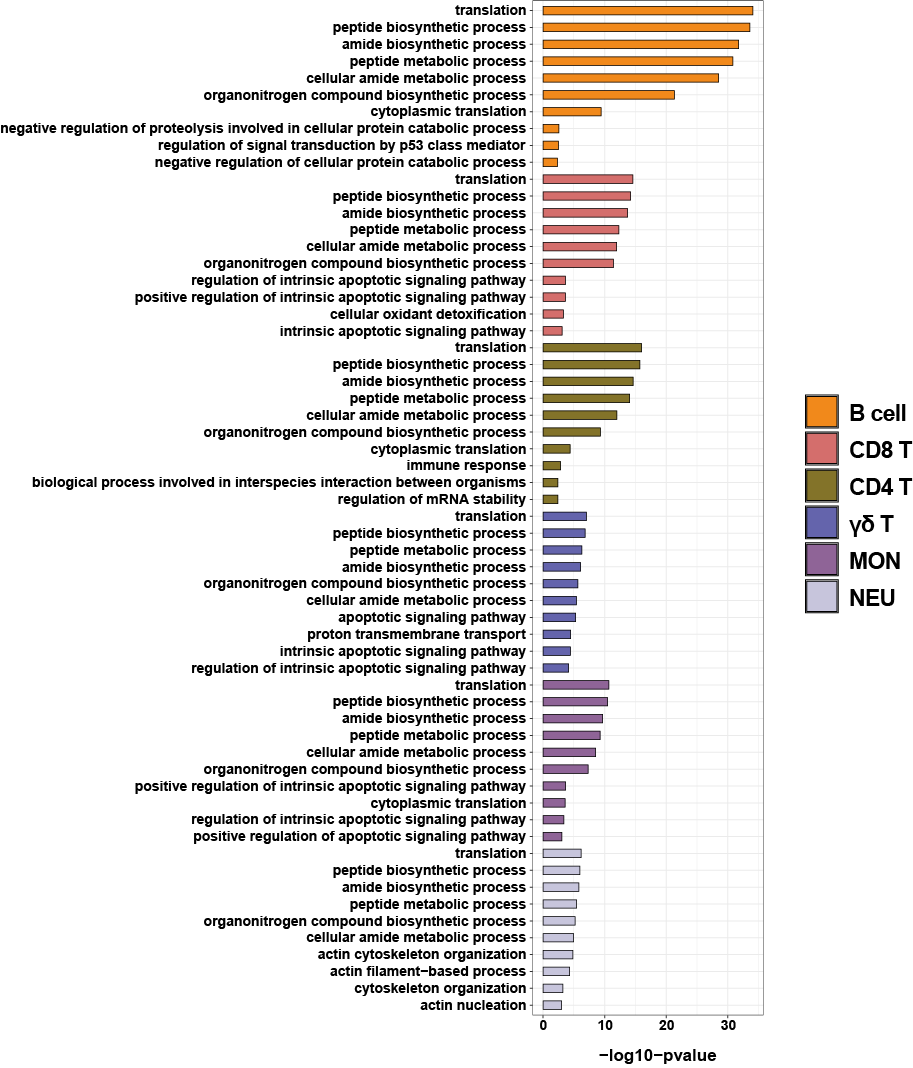


**Figure S2.** **Top ten representative immune biological pathways that enriched from the up-regulated differential expressed genes of immune cells isolated from HNF cow.** Pathways are presented as log10 p-value and color scheme is used to indicate immune cell population. MON: monocyte; NEU: neutrophil.


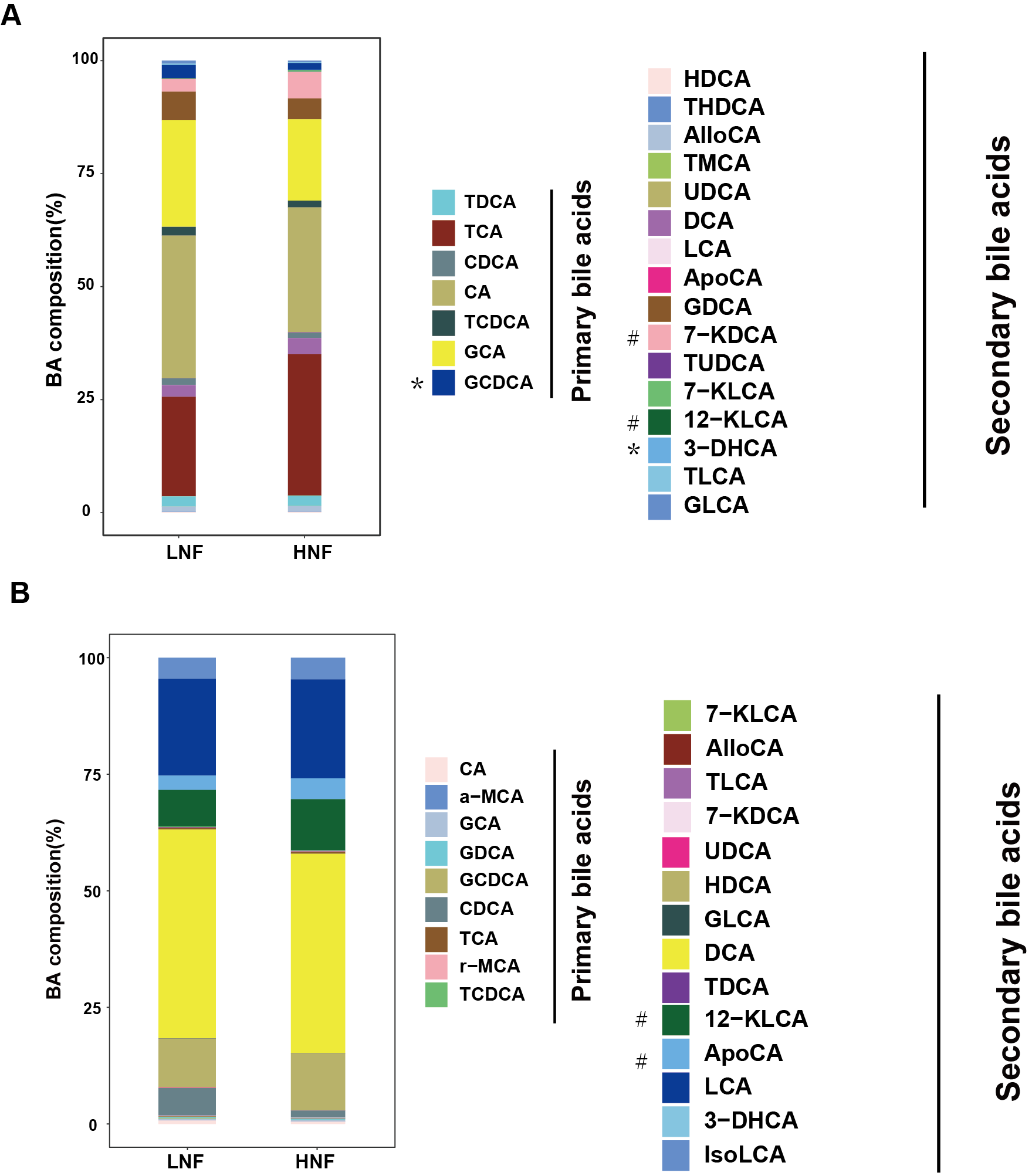


**Figure S3. The bile acid profile in plasma and feces of cows with low (LNF) and high lipolysis (HNF). A.** The percentage of plasma bile acid in two groups. **B.** The percentage of fecal bile acid in two groups. TDCA: Taurodeoxycholic acid; TCA: Taurocholic acid; CDCA: Chenodeoxycholic acid; CA: Cholic acid; TCDCA: Taurochenodeoxycholic acid; GCA: Glycocholic acid; GCDCA: Glycochenodeoxycholic acid; HDCA: Hyodeoxycholic acid; THDCA: Taurohyodeoxycholic acid; AlloCA: Allocholic acid; UDCA: Ursodeoxycholic acid; DCA: Deoxycholic acid; LCA: Lithocholic acid; ApoCA: Apocholic acid; GDCA: Glycodeoxycholic acid; 7-KDCA: 7-ketodeoxycholic acid; 7-KLCA: 7-Ketolithocholic acid; 12-KLCA: 12-ketolithocholic acid; 3-DHCA: 3-dehydrocholic acid; TLCA: Taurolithocholic acid; GLCA: Glycolithocholic acid; Total α-MCA: α-Muricholic acid; γ-MCA: γ-muricholic acid; isoLCA: Isolithocholic acid; TMCA: Tauro-muricholic acid; TUDCA: Tauroursodeoxycholic acid. LNF: cows with low lipolysis; HNF: cows with high lipolysis. * *P*-value < 0.05; # 0.05 < *P*-value < 0.10.

**
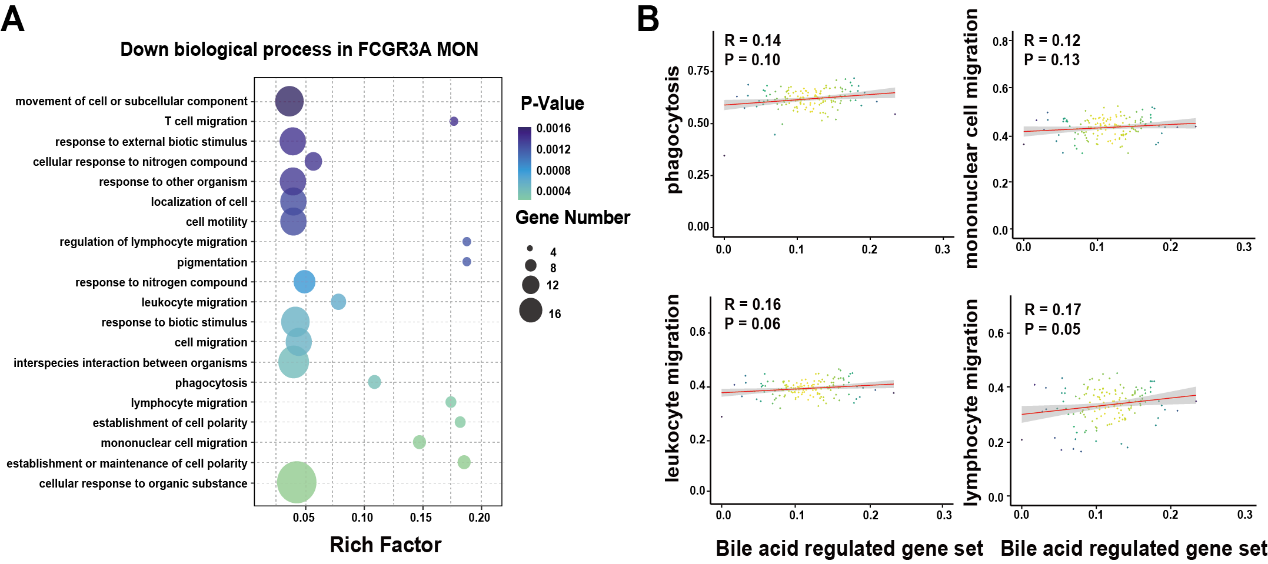
**

**Figure S4. The functional changes and associations with bile acid related gene set. A.** The enriched decreased biological process of FCGR3A^+^MON in HFNC compared to LFNC. **B.** Correlation of bile acid metabolism to the major decreased functions in FCGR3A^+^MON. LNFC: cow with low lipolysis; HNFC: cow with excessive lipolysis; MON: monocyte.
